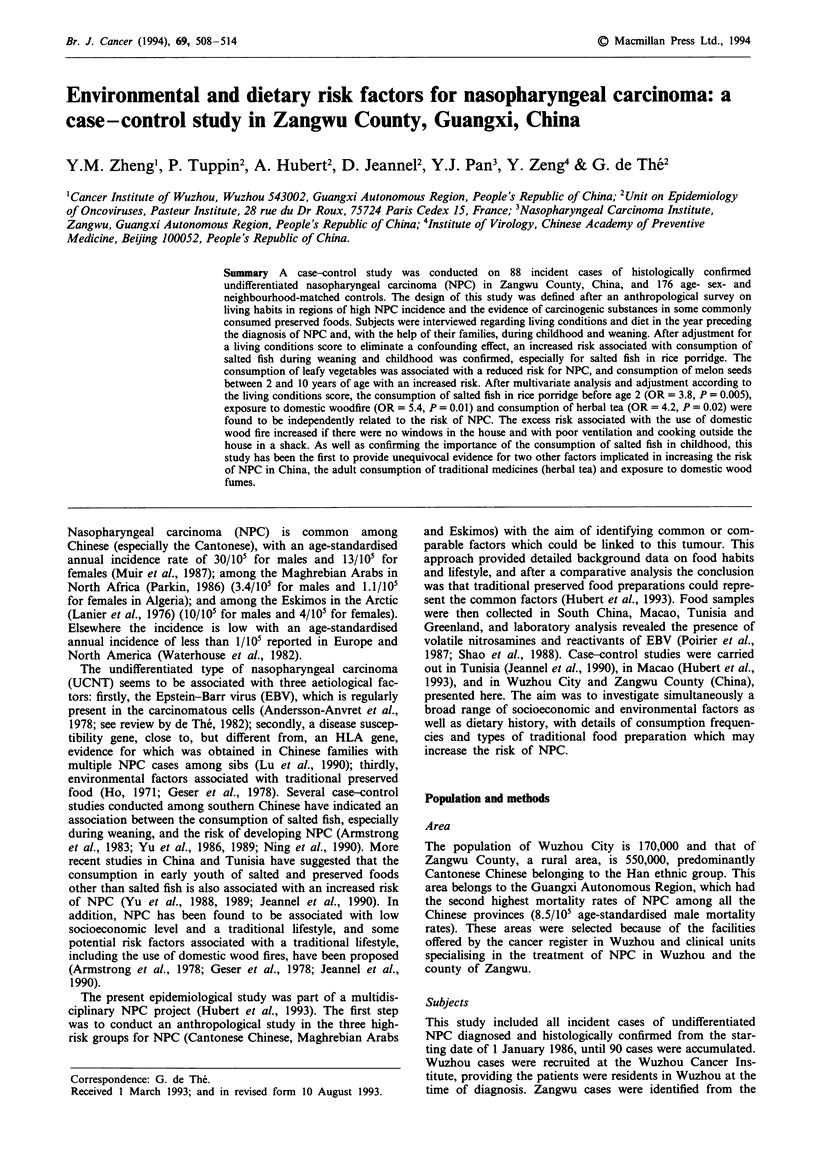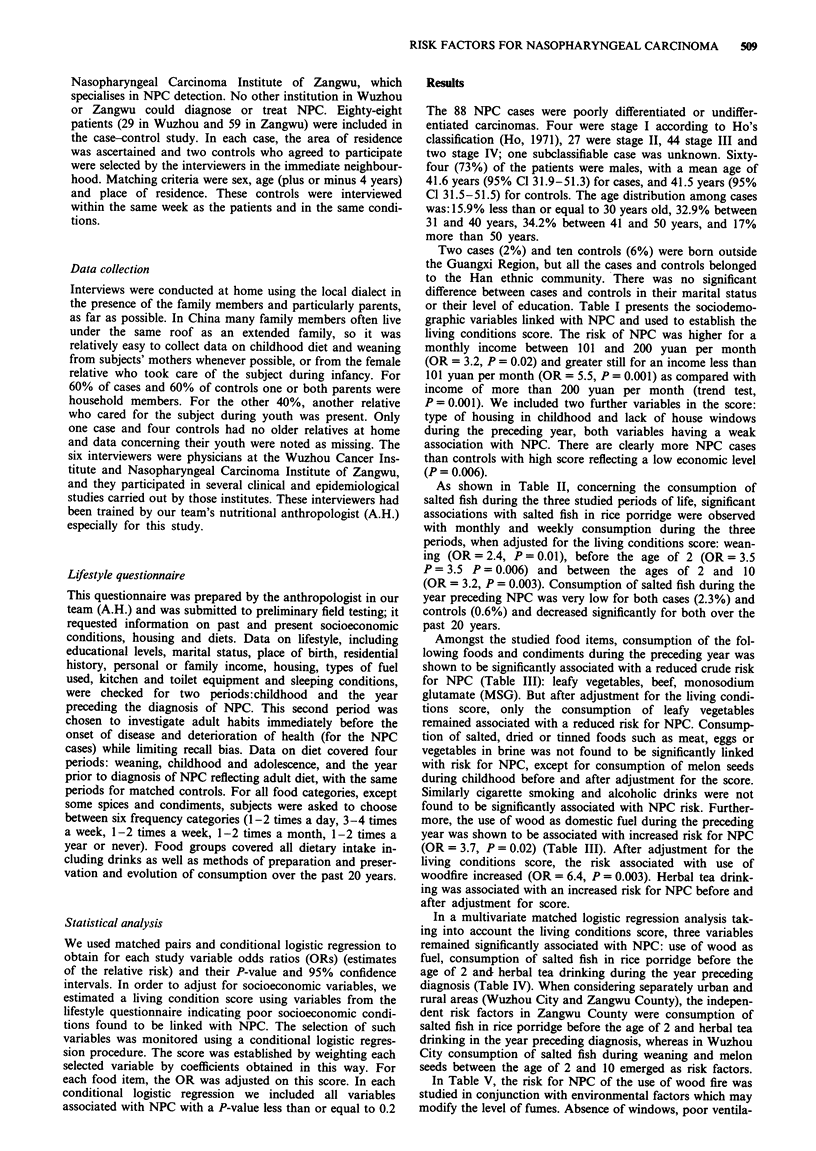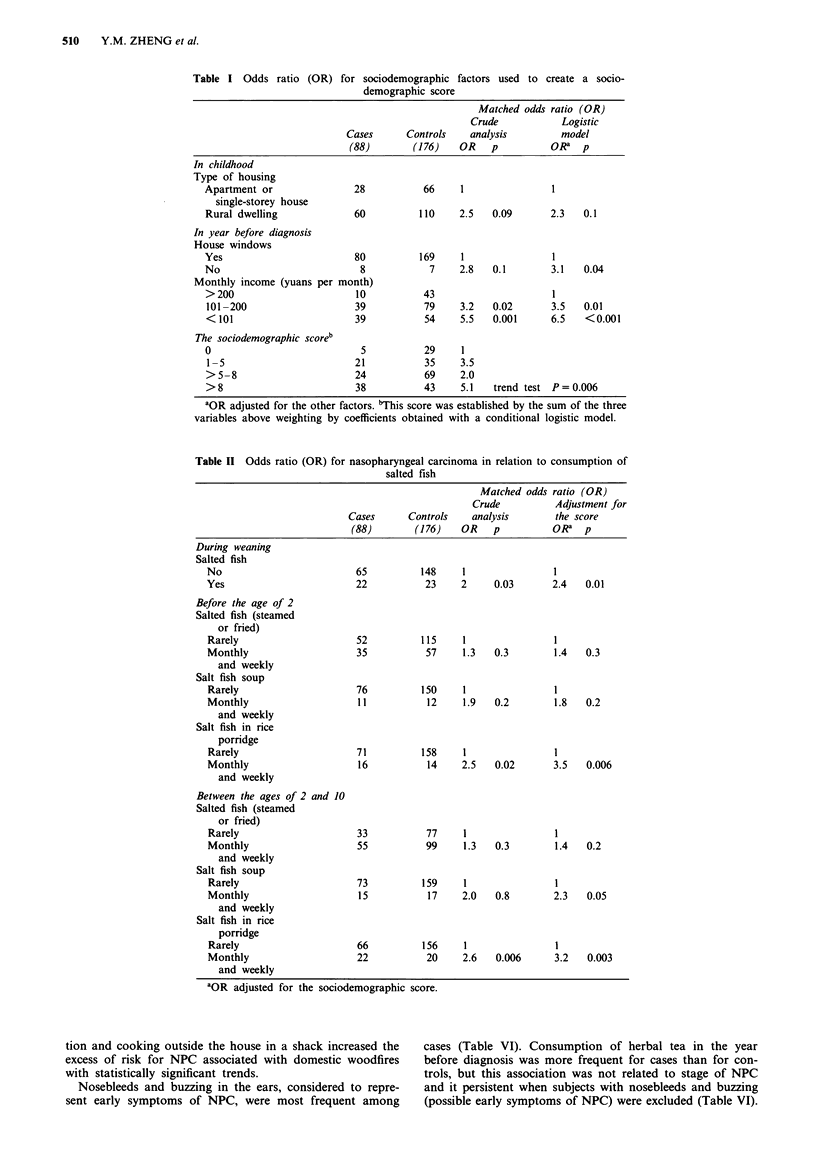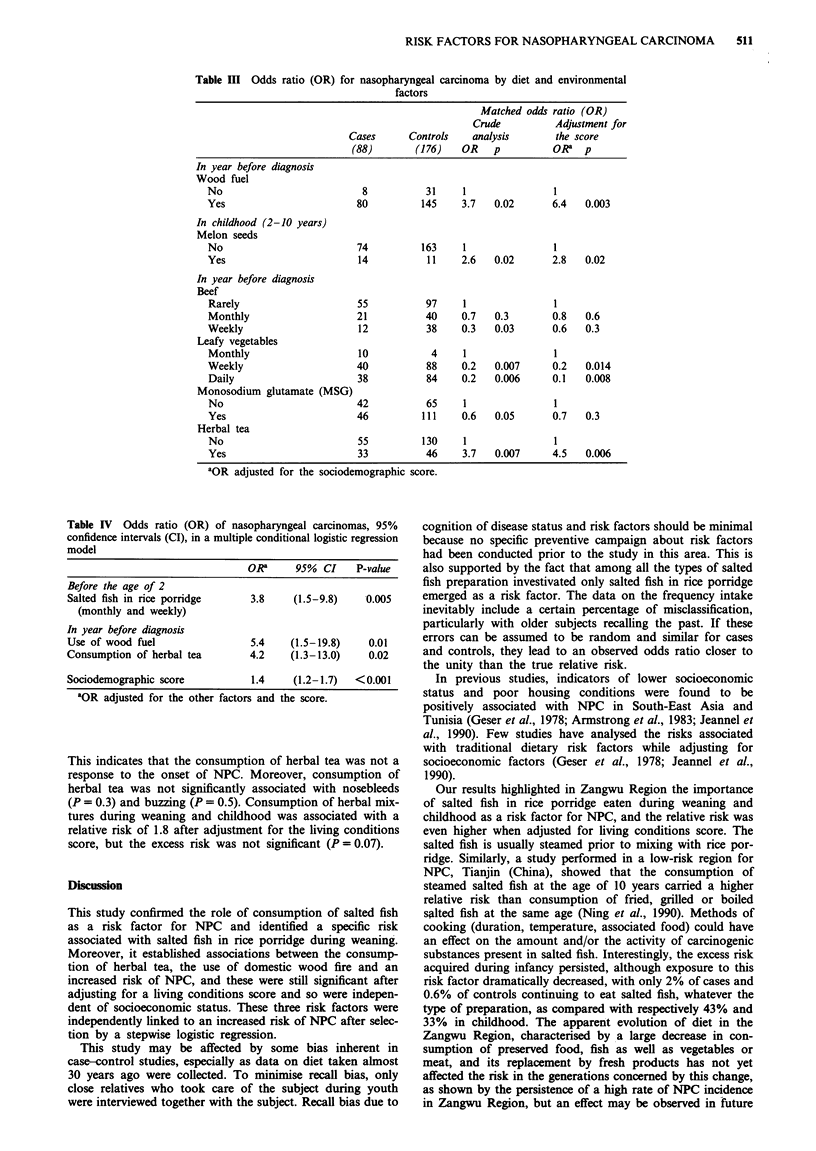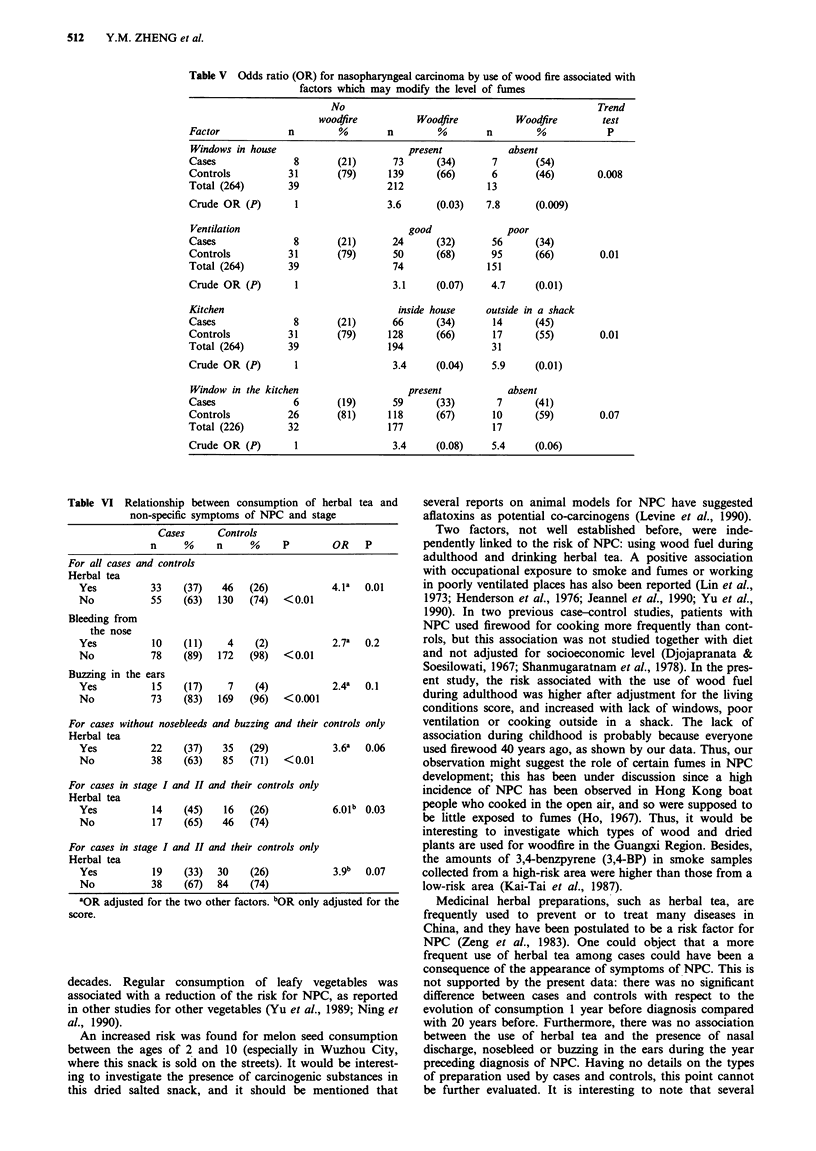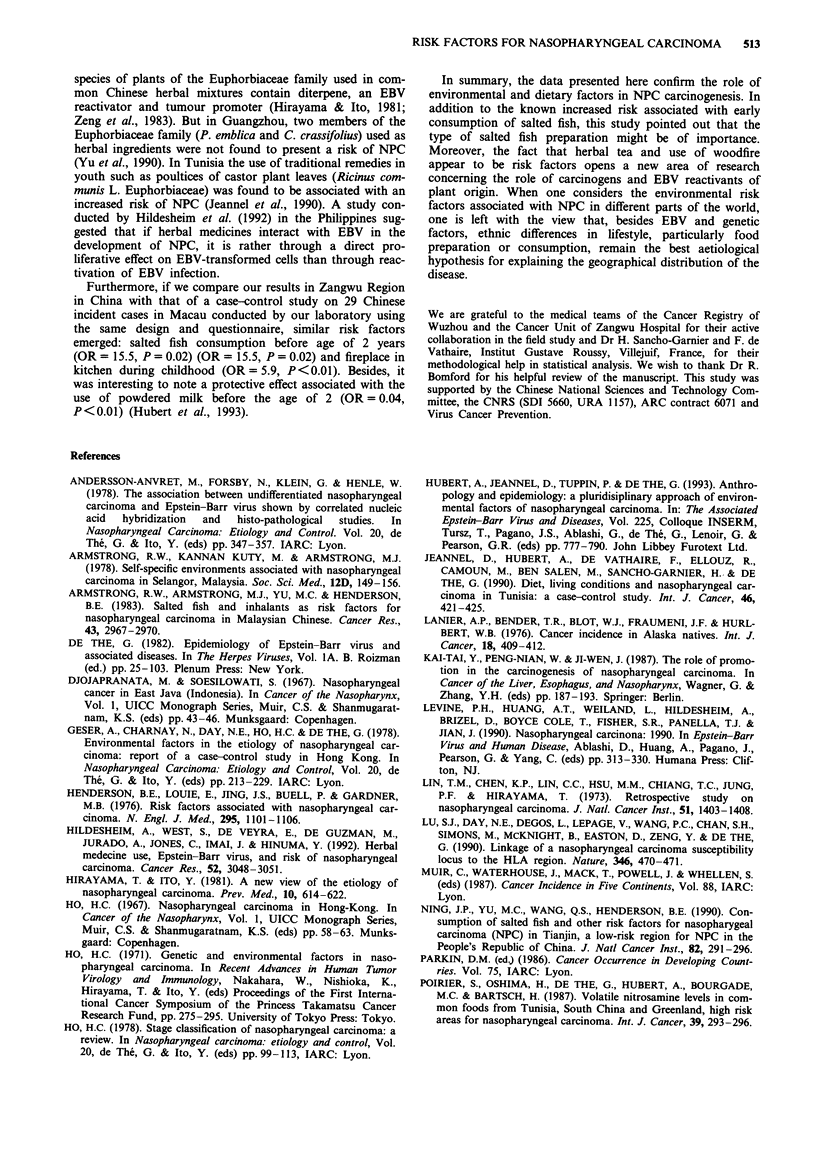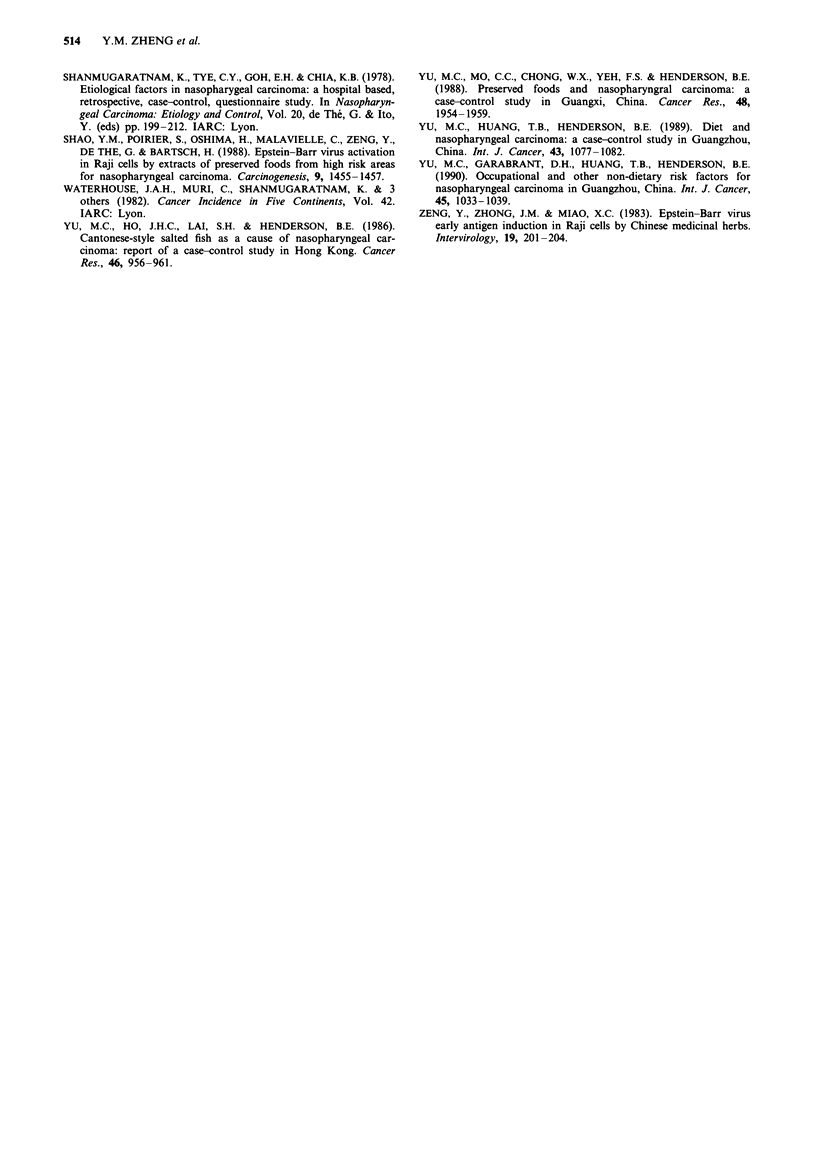# Environmental and dietary risk factors for nasopharyngeal carcinoma: a case-control study in Zangwu County, Guangxi, China.

**DOI:** 10.1038/bjc.1994.92

**Published:** 1994-03

**Authors:** Y. M. Zheng, P. Tuppin, A. Hubert, D. Jeannel, Y. J. Pan, Y. Zeng, G. de Thé

**Affiliations:** Cancer Institute of Wuzhou, Guangxi Autonomous Region, People's Republic of China.

## Abstract

A case-control study was conducted on 88 incident cases of histologically confirmed undifferentiated nasopharyngeal carcinoma (NPC) in Zangwu County, China, and 176 age- sex- and neighbourhood-matched controls. The design of this study was defined after an anthropological survey on living habits in regions of high NPC incidence and the evidence of carcinogenic substances in some commonly consumed preserved foods. Subjects were interviewed regarding living conditions and diet in the year preceding the diagnosis of NPC and, with the help of their families, during childhood and weaning. After adjustment for a living conditions score to eliminate a confounding effect, an increased risk associated with consumption of salted fish during weaning and childhood was confirmed, especially for salted fish in rice porridge. The consumption of leafy vegetables was associated with a reduced risk for NPC, and consumption of melon seeds between 2 and 10 years of age with an increased risk. After multivariate analysis and adjustment according to the living conditions score, the consumption of salted fish in rice porridge before age 2 (OR = 3.8, P = 0.005), exposure to domestic woodfire (OR = 5.4, P = 0.01) and consumption of herbal tea (OR = 4.2, P = 0.02) were found to be independently related to the risk of NPC. The excess risk associated with the use of domestic wood fire increased if there were no windows in the house and with poor ventilation and cooking outside the house in a shack. As well as confirming the importance of the consumption of salted fish in childhood, this study has been the first to provide unequivocal evidence for two other factors implicated in increasing the risk of NPC in China, the adult consumption of traditional medicines (herbal tea) and exposure to domestic wood fumes.


Br. J. Cancer (1994), 69, 508–514 © Macmillan Press Ltd., 1994

### Environmental and dietary risk factors for nasopharyngeal carcinoma: a case–control study in Zangwu County, Guangxi, China

Y.M. Zheng[1], P. Tuppin[2], A. Hubert[2], D. Jeannel[2], Y.J. Pan[3], Y. Zeng[4] & G. de Thé[2]

[1]*Cancer Institute of Wuzhou, Wuzhou 543002, Guangxi Autonomous Region, People's Republic of China;* [2]*Unit on Epidemiology of Oncoviruses, Pasteur Institute, 28 rue du Dr Roux, 75724 Paris Cedex 15, France;* [3]*Nasopharyngeal Carcinoma Institute, Zangwu, Guangxi Autonomous Region, People's Republic of China;* [4]*Institute of Virology, Chinese Academy of Preventive Medicine, Beijing 100052, People's Republic of China.*

**Summary** A case–control study was conducted on 88 incident cases of histologically confirmed undifferentiated nasopharyngeal carcinoma (NPC) in Zangwu County, China, and 176 age- sex- and neighbourhood-matched controls. The design of this study was defined after an anthropological survey on living habits in regions of high NPC incidence and the evidence of carcinogenic substances in some commonly consumed preserved foods. Subjects were interviewed regarding living conditions and diet in the year preceding the diagnosis of NPC and, with the help of their families, during childhood and weaning. After adjustment for a living conditions score to eliminate a confounding effect, an increased risk associated with consumption of salted fish during weaning and childhood was confirmed, especially for salted fish in rice porridge. The consumption of leafy vegetables was associated with a reduced risk for NPC, and consumption of melon seeds between 2 and 10 years of age with an increased risk. After multivariate analysis and adjustment according to the living conditions score, the consumption of salted fish in rice porridge before age 2 (OR = 3.8, $P = 0.005$), exposure to domestic woodfire (OR = 5.4, $P = 0.01$) and consumption of herbal tea (OR = 4.2, $P = 0.02$) were found to be independently related to the risk of NPC. The excess risk associated with the use of domestic wood fire increased if there were no windows in the house and with poor ventilation and cooking outside the house in a shack. As well as confirming the importance of the consumption of salted fish in childhood, this study has been the first to provide unequivocal evidence for two other factors implicated in increasing the risk of NPC in China, the adult consumption of traditional medicines (herbal tea) and exposure to domestic wood fumes.

Nasopharyngeal carcinoma (NPC) is common among Chinese (especially the Cantonese), with an age-standardised annual incidence rate of $30/10^5$ for males and $13/10^5$ for females (Muir *et al.*, 1987); among the Maghrebian Arabs in North Africa (Parkin, 1986) ($3.4/10^5$ for males and $1.1/10^5$ for females in Algeria); and among the Eskimos in the Arctic (Lanier *et al.*, 1976) ($10/10^5$ for males and $4/10^5$ for females). Elsewhere the incidence is low with an age-standardised annual incidence of less than $1/10^5$ reported in Europe and North America (Waterhouse *et al.*, 1982).

The undifferentiated type of nasopharyngeal carcinoma (UCNT) seems to be associated with three aetiological factors: firstly, the Epstein–Barr virus (EBV), which is regularly present in the carcinomatous cells (Andersson-Anvret *et al.*, 1978; see review by de Thé, 1982); secondly, a disease susceptibility gene, close to, but different from, an HLA gene, evidence for which was obtained in Chinese families with multiple NPC cases among sibs (Lu *et al.*, 1990); thirdly, environmental factors associated with traditional preserved food (Ho, 1971; Geser *et al.*, 1978). Several case–control studies conducted among southern Chinese have indicated an association between the consumption of salted fish, especially during weaning, and the risk of developing NPC (Armstrong *et al.*, 1983; Yu *et al.*, 1986, 1989; Ning *et al.*, 1990). More recent studies in China and Tunisia have suggested that the consumption in early youth of salted and preserved foods other than salted fish is also associated with an increased risk of NPC (Yu *et al.*, 1988, 1989; Jeannel *et al.*, 1990). In addition, NPC has been found to be associated with low socioeconomic level and a traditional lifestyle, and some potential risk factors associated with a traditional lifestyle, including the use of domestic wood fires, have been proposed (Armstrong *et al.*, 1978; Geser *et al.*, 1978; Jeannel *et al.*, 1990).

The present epidemiological study was part of a multidisciplinary NPC project (Hubert *et al.*, 1993). The first step was to conduct an anthropological study in the three high-risk groups for NPC (Cantonese Chinese, Maghrebian Arabs and Eskimos) with the aim of identifying common or comparable factors which could be linked to this tumour. This approach provided detailed background data on food habits and lifestyle, and after a comparative analysis the conclusion was that traditional preserved food preparations could represent the common factors (Hubert *et al.*, 1993). Food samples were then collected in South China, Macao, Tunisia and Greenland, and laboratory analysis revealed the presence of volatile nitrosamines and reactivants of EBV (Poirier *et al.*, 1987; Shao *et al.*, 1988). Case–control studies were carried out in Tunisia (Jeannel *et al.*, 1990), in Macao (Hubert *et al.*, 1993), and in Wuzhou City and Zangwu County (China), presented here. The aim was to investigate simultaneously a broad range of socioeconomic and environmental factors as well as dietary history, with details of consumption frequencies and types of traditional food preparation which may increase the risk of NPC.

### Population and methods

### *Area*

The population of Wuzhou City is 170,000 and that of Zangwu County, a rural area, is 550,000, predominantly Cantonese Chinese belonging to the Han ethnic group. This area belongs to the Guangxi Autonomous Region, which had the second highest mortality rates of NPC among all the Chinese provinces ($8.5/10^5$ age-standardised male mortality rates). These areas were selected because of the facilities offered by the cancer register in Wuzhou and clinical units specialising in the treatment of NPC in Wuzhou and the county of Zangwu.

### *Subjects*

This study included all incident cases of undifferentiated NPC diagnosed and histologically confirmed from the starting date of 1 January 1986, until 90 cases were accumulated. Wuzhou cases were recruited at the Wuzhou Cancer Institute, providing the patients were residents in Wuzhou at the time of diagnosis. Zangwu cases were identified from the

Correspondence: G. de Thé.
Received 1 March 1993; and in revised form 10 August 1993.

Nasopharyngeal Carcinoma Institute of Zangwu, which specialises in NPC detection. No other institution in Wuzhou or Zangwu could diagnose or treat NPC. Eighty-eight patients (29 in Wuzhou and 59 in Zangwu) were included in the case–control study. In each case, the area of residence was ascertained and two controls who agreed to participate were selected by the interviewers in the immediate neighbourhood. Matching criteria were sex, age (plus or minus 4 years) and place of residence. These controls were interviewed within the same week as the patients and in the same conditions.

*Data collection*

Interviews were conducted at home using the local dialect in the presence of the family members and particularly parents, as far as possible. In China many family members often live under the same roof as an extended family, so it was relatively easy to collect data on childhood diet and weaning from subjects' mothers whenever possible, or from the female relative who took care of the subject during infancy. For 60% of cases and 60% of controls one or both parents were household members. For the other 40%, another relative who cared for the subject during youth was present. Only one case and four controls had no older relatives at home and data concerning their youth were noted as missing. The six interviewers were physicians at the Wuzhou Cancer Institute and Nasopharyngeal Carcinoma Institute of Zangwu, and they participated in several clinical and epidemiological studies carried out by those institutes. These interviewers had been trained by our team's nutritional anthropologist (A.H.) especially for this study.

*Lifestyle questionnaire*

This questionnaire was prepared by the anthropologist in our team (A.H.) and was submitted to preliminary field testing; it requested information on past and present socioeconomic conditions, housing and diets. Data on lifestyle, including educational levels, marital status, place of birth, residential history, personal or family income, housing, types of fuel used, kitchen and toilet equipment and sleeping conditions, were checked for two periods:childhood and the year preceding the diagnosis of NPC. This second period was chosen to investigate adult habits immediately before the onset of disease and deterioration of health (for the NPC cases) while limiting recall bias. Data on diet covered four periods: weaning, childhood and adolescence, and the year prior to diagnosis of NPC reflecting adult diet, with the same periods for matched controls. For all food categories, except some spices and condiments, subjects were asked to choose between six frequency categories (1–2 times a day, 3–4 times a week, 1–2 times a week, 1–2 times a month, 1–2 times a year or never). Food groups covered all dietary intake including drinks as well as methods of preparation and preservation and evolution of consumption over the past 20 years.

*Statistical analysis*

We used matched pairs and conditional logistic regression to obtain for each study variable odds ratios (ORs) (estimates of the relative risk) and their *P*-value and 95% confidence intervals. In order to adjust for socioeconomic variables, we estimated a living condition score using variables from the lifestyle questionnaire indicating poor socioeconomic conditions found to be linked with NPC. The selection of such variables was monitored using a conditional logistic regression procedure. The score was established by weighting each selected variable by coefficients obtained in this way. For each food item, the OR was adjusted on this score. In each conditional logistic regression we included all variables associated with NPC with a *P*-value less than or equal to 0.2

**Results**

The 88 NPC cases were poorly differentiated or undifferentiated carcinomas. Four were stage I according to Ho's classification (Ho, 1971), 27 were stage II, 44 stage III and two stage IV; one subclassifiable case was unknown. Sixty-four (73%) of the patients were males, with a mean age of 41.6 years (95% Cl 31.9–51.3) for cases, and 41.5 years (95% Cl 31.5–51.5) for controls. The age distribution among cases was:15.9% less than or equal to 30 years old, 32.9% between 31 and 40 years, 34.2% between 41 and 50 years, and 17% more than 50 years.

Two cases (2%) and ten controls (6%) were born outside the Guangxi Region, but all the cases and controls belonged to the Han ethnic community. There was no significant difference between cases and controls in their marital status or their level of education. Table I presents the sociodemographic variables linked with NPC and used to establish the living conditions score. The risk of NPC was higher for a monthly income between 101 and 200 yuan per month (OR = 3.2, $P = 0.02$) and greater still for an income less than 101 yuan per month (OR = 5.5, $P = 0.001$) as compared with income of more than 200 yuan per month (trend test, $P = 0.001$). We included two further variables in the score: type of housing in childhood and lack of house windows during the preceding year, both variables having a weak association with NPC. There are clearly more NPC cases than controls with high score reflecting a low economic level ($P = 0.006$).

As shown in Table II, concerning the consumption of salted fish during the three studied periods of life, significant associations with salted fish in rice porridge were observed with monthly and weekly consumption during the three periods, when adjusted for the living conditions score: weaning (OR = 2.4, $P = 0.01$), before the age of 2 (OR = 3.5 $P = 3.5$ $P = 0.006$) and between the ages of 2 and 10 (OR = 3.2, $P = 0.003$). Consumption of salted fish during the year preceding NPC was very low for both cases (2.3%) and controls (0.6%) and decreased significantly for both over the past 20 years.

Amongst the studied food items, consumption of the following foods and condiments during the preceding year was shown to be significantly associated with a reduced crude risk for NPC (Table III): leafy vegetables, beef, monosodium glutamate (MSG). But after adjustment for the living conditions score, only the consumption of leafy vegetables remained associated with a reduced risk for NPC. Consumption of salted, dried or tinned foods such as meat, eggs or vegetables in brine was not found to be significantly linked with risk for NPC, except for consumption of melon seeds during childhood before and after adjustment for the score. Similarly cigarette smoking and alcoholic drinks were not found to be significantly associated with NPC risk. Furthermore, the use of wood as domestic fuel during the preceding year was shown to be associated with increased risk for NPC (OR = 3.7, $P = 0.02$) (Table III). After adjustment for the living conditions score, the risk associated with use of woodfire increased (OR = 6.4, $P = 0.003$). Herbal tea drinking was associated with an increased risk for NPC before and after adjustment for score.

In a multivariate matched logistic regression analysis taking into account the living conditions score, three variables remained significantly associated with NPC: use of wood as fuel, consumption of salted fish in rice porridge before the age of 2 and herbal tea drinking during the year preceding diagnosis (Table IV). When considering separately urban and rural areas (Wuzhou City and Zangwu County), the independent risk factors in Zangwu County were consumption of salted fish in rice porridge before the age of 2 and herbal tea drinking in the year preceding diagnosis, whereas in Wuzhou City consumption of salted fish during weaning and melon seeds between the age of 2 and 10 emerged as risk factors.

In Table V, the risk for NPC of the use of wood fire was studied in conjunction with environmental factors which may modify the level of fumes. Absence of windows, poor ventila-

**Table I** Odds ratio (OR) for sociodemographic factors used to create a sociodemographic score

| | Cases (88) | Controls (176) | Matched odds ratio (OR) Crude analysis OR | p | Logistic model OR[a] | p |
|---|---|---|---|---|---|---|
| *In childhood* | | | | | | |
| Type of housing | | | | | | |
| Apartment or single-storey house | 28 | 66 | 1 | | 1 | |
| Rural dwelling | 60 | 110 | 2.5 | 0.09 | 2.3 | 0.1 |
| *In year before diagnosis* | | | | | | |
| House windows | | | | | | |
| Yes | 80 | 169 | 1 | | 1 | |
| No | 8 | 7 | 2.8 | 0.1 | 3.1 | 0.04 |
| Monthly income (yuans per month) | | | | | | |
| >200 | 10 | 43 | | | 1 | |
| 101–200 | 39 | 79 | 3.2 | 0.02 | 3.5 | 0.01 |
| <101 | 39 | 54 | 5.5 | 0.001 | 6.5 | <0.001 |
| *The sociodemographic score*[b] | | | | | | |
| 0 | 5 | 29 | 1 | | | |
| 1–5 | 21 | 35 | 3.5 | | | |
| >5–8 | 24 | 69 | 2.0 | | | |
| >8 | 38 | 43 | 5.1 | trend test | $P = 0.006$ | |

[a]OR adjusted for the other factors. [b]This score was established by the sum of the three variables above weighting by coefficients obtained with a conditional logistic model.

**Table II** Odds ratio (OR) for nasopharyngeal carcinoma in relation to consumption of salted fish

| | Cases (88) | Controls (176) | Matched odds ratio (OR) Crude analysis OR | p | Adjustment for the score OR[a] | p |
|---|---|---|---|---|---|---|
| *During weaning* | | | | | | |
| Salted fish | | | | | | |
| No | 65 | 148 | 1 | | 1 | |
| Yes | 22 | 23 | 2 | 0.03 | 2.4 | 0.01 |
| *Before the age of 2* | | | | | | |
| Salted fish (steamed or fried) | | | | | | |
| Rarely | 52 | 115 | 1 | | 1 | |
| Monthly and weekly | 35 | 57 | 1.3 | 0.3 | 1.4 | 0.3 |
| Salt fish soup | | | | | | |
| Rarely | 76 | 150 | 1 | | 1 | |
| Monthly and weekly | 11 | 12 | 1.9 | 0.2 | 1.8 | 0.2 |
| Salt fish in rice porridge | | | | | | |
| Rarely | 71 | 158 | 1 | | 1 | |
| Monthly and weekly | 16 | 14 | 2.5 | 0.02 | 3.5 | 0.006 |
| *Between the ages of 2 and 10* | | | | | | |
| Salted fish (steamed or fried) | | | | | | |
| Rarely | 33 | 77 | 1 | | 1 | |
| Monthly and weekly | 55 | 99 | 1.3 | 0.3 | 1.4 | 0.2 |
| Salt fish soup | | | | | | |
| Rarely | 73 | 159 | 1 | | 1 | |
| Monthly and weekly | 15 | 17 | 2.0 | 0.8 | 2.3 | 0.05 |
| Salt fish in rice porridge | | | | | | |
| Rarely | 66 | 156 | 1 | | 1 | |
| Monthly and weekly | 22 | 20 | 2.6 | 0.006 | 3.2 | 0.003 |

[a]OR adjusted for the sociodemographic score.

tion and cooking outside the house in a shack increased the excess of risk for NPC associated with domestic woodfires with statistically significant trends.

Nosebleeds and buzzing in the ears, considered to represent early symptoms of NPC, were most frequent among cases (Table VI). Consumption of herbal tea in the year before diagnosis was more frequent for cases than for controls, but this association was not related to stage of NPC and it persistent when subjects with nosebleeds and buzzing (possible early symptoms of NPC) were excluded (Table VI).

**Table III** Odds ratio (OR) for nasopharyngeal carcinoma by diet and environmental factors

| | Cases (88) | Controls (176) | Matched odds ratio (OR) Crude analysis OR | p | Adjustment for the score OR[a] | p |
|---|---|---|---|---|---|---|
| *In year before diagnosis* | | | | | | |
| Wood fuel | | | | | | |
| No | 8 | 31 | 1 | | 1 | |
| Yes | 80 | 145 | 3.7 | 0.02 | 6.4 | 0.003 |
| *In childhood (2–10 years)* | | | | | | |
| Melon seeds | | | | | | |
| No | 74 | 163 | 1 | | 1 | |
| Yes | 14 | 11 | 2.6 | 0.02 | 2.8 | 0.02 |
| *In year before diagnosis* | | | | | | |
| Beef | | | | | | |
| Rarely | 55 | 97 | 1 | | 1 | |
| Monthly | 21 | 40 | 0.7 | 0.3 | 0.8 | 0.6 |
| Weekly | 12 | 38 | 0.3 | 0.03 | 0.6 | 0.3 |
| Leafy vegetables | | | | | | |
| Monthly | 10 | 4 | 1 | | 1 | |
| Weekly | 40 | 88 | 0.2 | 0.007 | 0.2 | 0.014 |
| Daily | 38 | 84 | 0.2 | 0.006 | 0.1 | 0.008 |
| Monosodium glutamate (MSG) | | | | | | |
| No | 42 | 65 | 1 | | 1 | |
| Yes | 46 | 111 | 0.6 | 0.05 | 0.7 | 0.3 |
| Herbal tea | | | | | | |
| No | 55 | 130 | 1 | | 1 | |
| Yes | 33 | 46 | 3.7 | 0.007 | 4.5 | 0.006 |

[a]OR adjusted for the sociodemographic score.

**Table IV** Odds ratio (OR) of nasopharyngeal carcinomas, 95% confidence intervals (CI), in a multiple conditional logistic regression model

| | OR[a] | 95% CI | P-value |
|---|---|---|---|
| *Before the age of 2* | | | |
| Salted fish in rice porridge (monthly and weekly) | 3.8 | (1.5–9.8) | 0.005 |
| *In year before diagnosis* | | | |
| Use of wood fuel | 5.4 | (1.5–19.8) | 0.01 |
| Consumption of herbal tea | 4.2 | (1.3–13.0) | 0.02 |
| Sociodemographic score | 1.4 | (1.2–1.7) | <0.001 |

[a]OR adjusted for the other factors and the score.

This indicates that the consumption of herbal tea was not a response to the onset of NPC. Moreover, consumption of herbal tea was not significantly associated with nosebleeds ($P = 0.3$) and buzzing ($P = 0.5$). Consumption of herbal mixtures during weaning and childhood was associated with a relative risk of 1.8 after adjustment for the living conditions score, but the excess risk was not significant ($P = 0.07$).

### Discussion

This study confirmed the role of consumption of salted fish as a risk factor for NPC and identified a specific risk associated with salted fish in rice porridge during weaning. Moreover, it established associations between the consumption of herbal tea, the use of domestic wood fire and an increased risk of NPC, and these were still significant after adjusting for a living conditions score and so were independent of socioeconomic status. These three risk factors were independently linked to an increased risk of NPC after selection by a stepwise logistic regression.

This study may be affected by some bias inherent in case–control studies, especially as data on diet taken almost 30 years ago were collected. To minimise recall bias, only close relatives who took care of the subject during youth were interviewed together with the subject. Recall bias due to cognition of disease status and risk factors should be minimal because no specific preventive campaign about risk factors had been conducted prior to the study in this area. This is also supported by the fact that among all the types of salted fish preparation investivated only salted fish in rice porridge emerged as a risk factor. The data on the frequency intake inevitably include a certain percentage of misclassification, particularly with older subjects recalling the past. If these errors can be assumed to be random and similar for cases and controls, they lead to an observed odds ratio closer to the unity than the true relative risk.

In previous studies, indicators of lower socioeconomic status and poor housing conditions were found to be positively associated with NPC in South-East Asia and Tunisia (Geser *et al.*, 1978; Armstrong *et al.*, 1983; Jeannel *et al.*, 1990). Few studies have analysed the risks associated with traditional dietary risk factors while adjusting for socioeconomic factors (Geser *et al.*, 1978; Jeannel *et al.*, 1990).

Our results highlighted in Zangwu Region the importance of salted fish in rice porridge eaten during weaning and childhood as a risk factor for NPC, and the relative risk was even higher when adjusted for living conditions score. The salted fish is usually steamed prior to mixing with rice porridge. Similarly, a study performed in a low-risk region for NPC, Tianjin (China), showed that the consumption of steamed salted fish at the age of 10 years carried a higher relative risk than consumption of fried, grilled or boiled salted fish at the same age (Ning *et al.*, 1990). Methods of cooking (duration, temperature, associated food) could have an effect on the amount and/or the activity of carcinogenic substances present in salted fish. Interestingly, the excess risk acquired during infancy persisted, although exposure to this risk factor dramatically decreased, with only 2% of cases and 0.6% of controls continuing to eat salted fish, whatever the type of preparation, as compared with respectively 43% and 33% in childhood. The apparent evolution of diet in the Zangwu Region, characterised by a large decrease in consumption of preserved food, fish as well as vegetables or meat, and its replacement by fresh products has not yet affected the risk in the generations concerned by this change, as shown by the persistence of a high rate of NPC incidence in Zangwu Region, but an effect may be observed in future

**Table V** Odds ratio (OR) for nasopharyngeal carcinoma by use of wood fire associated with factors which may modify the level of fumes

| *Factor* | n | *No woodfire* % | n | *Woodfire* % | n | *Woodfire* % | *Trend test* P |
|---|---|---|---|---|---|---|---|
| *Windows in house* | | | | *present* | | *absent* | |
| Cases | 8 | (21) | 73 | (34) | 7 | (54) | |
| Controls | 31 | (79) | 139 | (66) | 6 | (46) | 0.008 |
| Total (264) | 39 | | 212 | | 13 | | |
| Crude OR (*P*) | 1 | | 3.6 | (0.03) | 7.8 | (0.009) | |
| *Ventilation* | | | | *good* | | *poor* | |
| Cases | 8 | (21) | 24 | (32) | 56 | (34) | |
| Controls | 31 | (79) | 50 | (68) | 95 | (66) | 0.01 |
| Total (264) | 39 | | 74 | | 151 | | |
| Crude OR (*P*) | 1 | | 3.1 | (0.07) | 4.7 | (0.01) | |
| *Kitchen* | | | | *inside house* | | *outside in a shack* | |
| Cases | 8 | (21) | 66 | (34) | 14 | (45) | |
| Controls | 31 | (79) | 128 | (66) | 17 | (55) | 0.01 |
| Total (264) | 39 | | 194 | | 31 | | |
| Crude OR (*P*) | 1 | | 3.4 | (0.04) | 5.9 | (0.01) | |
| *Window in the kitchen* | | | | *present* | | *absent* | |
| Cases | 6 | (19) | 59 | (33) | 7 | (41) | |
| Controls | 26 | (81) | 118 | (67) | 10 | (59) | 0.07 |
| Total (226) | 32 | | 177 | | 17 | | |
| Crude OR (*P*) | 1 | | 3.4 | (0.08) | 5.4 | (0.06) | |

**Table VI** Relationship between consumption of herbal tea and non-specific symptoms of NPC and stage

| | *Cases* n | *Cases* % | *Controls* n | *Controls* % | P | *OR* | P |
|---|---|---|---|---|---|---|---|
| *For all cases and controls* | | | | | | | |
| Herbal tea | | | | | | | |
| Yes | 33 | (37) | 46 | (26) | | 4.1[a] | 0.01 |
| No | 55 | (63) | 130 | (74) | <0.01 | | |
| Bleeding from the nose | | | | | | | |
| Yes | 10 | (11) | 4 | (2) | | 2.7[a] | 0.2 |
| No | 78 | (89) | 172 | (98) | <0.01 | | |
| Buzzing in the ears | | | | | | | |
| Yes | 15 | (17) | 7 | (4) | | 2.4[a] | 0.1 |
| No | 73 | (83) | 169 | (96) | <0.001 | | |
| *For cases without nosebleeds and buzzing and their controls only* | | | | | | | |
| Herbal tea | | | | | | | |
| Yes | 22 | (37) | 35 | (29) | | 3.6[a] | 0.06 |
| No | 38 | (63) | 85 | (71) | <0.01 | | |
| *For cases in stage I and II and their controls only* | | | | | | | |
| Herbal tea | | | | | | | |
| Yes | 14 | (45) | 16 | (26) | | 6.01[b] | 0.03 |
| No | 17 | (65) | 46 | (74) | | | |
| *For cases in stage I and II and their controls only* | | | | | | | |
| Herbal tea | | | | | | | |
| Yes | 19 | (33) | 30 | (26) | | 3.9[b] | 0.07 |
| No | 38 | (67) | 84 | (74) | | | |

[a]OR adjusted for the two other factors. [b]OR only adjusted for the score.

decades. Regular consumption of leafy vegetables was associated with a reduction of the risk for NPC, as reported in other studies for other vegetables (Yu *et al.*, 1989; Ning *et al.*, 1990).

An increased risk was found for melon seed consumption between the ages of 2 and 10 (especially in Wuzhou City, where this snack is sold on the streets). It would be interesting to investigate the presence of carcinogenic substances in this dried salted snack, and it should be mentioned that several reports on animal models for NPC have suggested aflatoxins as potential co-carcinogens (Levine *et al.*, 1990).

Two factors, not well established before, were independently linked to the risk of NPC: using wood fuel during adulthood and drinking herbal tea. A positive association with occupational exposure to smoke and fumes or working in poorly ventilated places has also been reported (Lin *et al.*, 1973; Henderson *et al.*, 1976; Jeannel *et al.*, 1990; Yu *et al.*, 1990). In two previous case–control studies, patients with NPC used firewood for cooking more frequently than controls, but this association was not studied together with diet and not adjusted for socioeconomic level (Djojapranata & Soesilowati, 1967; Shanmugaratnam *et al.*, 1978). In the present study, the risk associated with the use of wood fuel during adulthood was higher after adjustment for the living conditions score, and increased with lack of windows, poor ventilation or cooking outside in a shack. The lack of association during childhood is probably because everyone used firewood 40 years ago, as shown by our data. Thus, our observation might suggest the role of certain fumes in NPC development; this has been under discussion since a high incidence of NPC has been observed in Hong Kong boat people who cooked in the open air, and so were supposed to be little exposed to fumes (Ho, 1967). Thus, it would be interesting to investigate which types of wood and dried plants are used for woodfire in the Guangxi Region. Besides, the amounts of 3,4-benzpyrene (3,4-BP) in smoke samples collected from a high-risk area were higher than those from a low-risk area (Kai-Tai *et al.*, 1987).

Medicinal herbal preparations, such as herbal tea, are frequently used to prevent or to treat many diseases in China, and they have been postulated to be a risk factor for NPC (Zeng *et al.*, 1983). One could object that a more frequent use of herbal tea among cases could have been a consequence of the appearance of symptoms of NPC. This is not supported by the present data: there was no significant difference between cases and controls with respect to the evolution of consumption 1 year before diagnosis compared with 20 years before. Furthermore, there was no association between the use of herbal tea and the presence of nasal discharge, nosebleed or buzzing in the ears during the year preceding diagnosis of NPC. Having no details on the types of preparation used by cases and controls, this point cannot be further evaluated. It is interesting to note that several species of plants of the Euphorbiaceae family used in common Chinese herbal mixtures contain diterpene, an EBV reactivator and tumour promoter (Hirayama & Ito, 1981; Zeng *et al.*, 1983). But in Guangzhou, two members of the Euphorbiaceae family (*P. emblica* and *C. crassifolius*) used as herbal ingredients were not found to present a risk of NPC (Yu *et al.*, 1990). In Tunisia the use of traditional remedies in youth such as poultices of castor plant leaves (*Ricinus communis* L. Euphorbiaceae) was found to be associated with an increased risk of NPC (Jeannel *et al.*, 1990). A study conducted by Hildesheim *et al.* (1992) in the Philippines suggested that if herbal medicines interact with EBV in the development of NPC, it is rather through a direct proliferative effect on EBV-transformed cells than through reactivation of EBV infection.

Furthermore, if we compare our results in Zangwu Region in China with that of a case–control study on 29 Chinese incident cases in Macau conducted by our laboratory using the same design and questionnaire, similar risk factors emerged: salted fish consumption before age of 2 years (OR = 15.5, $P = 0.02$) (OR = 15.5, $P = 0.02$) and fireplace in kitchen during childhood (OR = 5.9, $P < 0.01$). Besides, it was interesting to note a protective effect associated with the use of powdered milk before the age of 2 (OR = 0.04, $P < 0.01$) (Hubert *et al.*, 1993).

In summary, the data presented here confirm the role of environmental and dietary factors in NPC carcinogenesis. In addition to the known increased risk associated with early consumption of salted fish, this study pointed out that the type of salted fish preparation might be of importance. Moreover, the fact that herbal tea and use of woodfire appear to be risk factors opens a new area of research concerning the role of carcinogens and EBV reactivants of plant origin. When one considers the environmental risk factors associated with NPC in different parts of the world, one is left with the view that, besides EBV and genetic factors, ethnic differences in lifestyle, particularly food preparation or consumption, remain the best aetiological hypothesis for explaining the geographical distribution of the disease.

We are grateful to the medical teams of the Cancer Registry of Wuzhou and the Cancer Unit of Zangwu Hospital for their active collaboration in the field study and Dr H. Sancho-Garnier and F. de Vathaire, Institut Gustave Roussy, Villejuif, France, for their methodological help in statistical analysis. We wish to thank Dr R. Bomford for his helpful review of the manuscript. This study was supported by the Chinese National Sciences and Technology Committee, the CNRS (SDI 5660, URA 1157), ARC contract 6071 and Virus Cancer Prevention.

### References

ANDERSSON-ANVRET, M., FORSBY, N., KLEIN, G. & HENLE, W. (1978). The association between undifferentiated nasopharyngeal carcinoma and Epstein–Barr virus shown by correlated nucleic acid hybridization and histo-pathological studies. In *Nasopharyngeal Carcinoma: Etiology and Control.* Vol. 20, de Thé, G. & Ito, Y. (eds) pp. 347–357. IARC: Lyon.

ARMSTRONG, R.W., KANNAN KUTY, M. & ARMSTRONG, M.J. (1978). Self-specific environments associated with nasopharyngeal carcinoma in Selangor, Malaysia. *Soc. Sci. Med.*, **12D**, 149–156.

ARMSTRONG, R.W., ARMSTRONG, M.J., YU, M.C. & HENDERSON, B.E. (1983). Salted fish and inhalants as risk factors for nasopharyngeal carcinoma in Malaysian Chinese. *Cancer Res.*, **43**, 2967–2970.

DE THE, G. (1982). Epidemiology of Epstein–Barr virus and associated diseases. In *The Herpes Viruses*, Vol. 1A. B. Roizman (ed.) pp. 25–103. Plenum Press: New York.

DJOJAPRANATA, M. & SOESILOWATI, S. (1967). Nasopharyngeal cancer in East Java (Indonesia). In *Cancer of the Nasopharynx*, Vol. 1, UICC Monograph Series, Muir, C.S. & Shanmugaratnam, K.S. (eds) pp. 43–46. Munksgaard: Copenhagen.

GESER, A., CHARNAY, N., DAY, N.E., HO, H.C. & DE THE, G. (1978). Environmental factors in the etiology of nasopharyngeal carcinoma: report of a case–control study in Hong Kong. In *Nasopharyngeal Carcinoma: Etiology and Control*, Vol. 20, de Thé, G. & Ito, Y. (eds) pp. 213–229. IARC: Lyon.

HENDERSON, B.E., LOUIE, E., JING, J.S., BUELL, P. & GARDNER, M.B. (1976). Risk factors associated with nasopharyngeal carcinoma. *N. Engl. J. Med.*, **295**, 1101–1106.

HILDESHEIM, A., WEST, S., DE VEYRA, E., DE GUZMAN, M., JURADO, A., JONES, C., IMAI, J. & HINUMA, Y. (1992). Herbal medecine use, Epstein–Barr virus, and risk of nasopharyngeal carcinoma. *Cancer Res.*, **52**, 3048–3051.

HIRAYAMA, T. & ITO, Y. (1981). A new view of the etiology of nasopharyngeal carcinoma. *Prev. Med.*, **10**, 614–622.

HO, H.C. (1967). Nasopharyngeal carcinoma in Hong-Kong. In *Cancer of the Nasopharynx*, Vol. 1, UICC Monograph Series, Muir, C.S. & Shanmugaratnam, K.S. (eds) pp. 58–63. Munksgaard: Copenhagen.

HO, H.C. (1971). Genetic and environmental factors in nasopharyngeal carcinoma. In *Recent Advances in Human Tumor Virology and Immunology*, Nakahara, W., Nishioka, K., Hirayama, T. & Ito, Y. (eds) Proceedings of the First International Cancer Symposium of the Princess Takamatsu Cancer Research Fund, pp. 275–295. University of Tokyo Press: Tokyo.

HO, H.C. (1978). Stage classification of nasopharyngeal carcinoma: a review. In *Nasopharyngeal carcinoma: etiology and control*, Vol. 20, de Thé, G. & Ito, Y. (eds) pp. 99–113, IARC: Lyon.

HUBERT, A., JEANNEL, D., TUPPIN, P. & DE THE, G. (1993). Anthropology and epidemiology: a pluridisiplinary approach of environmental factors of nasopharyngeal carcinoma. In: *The Associated Epstein–Barr Virus and Diseases*, Vol. 225, Colloque INSERM, Tursz, T., Pagano, J.S., Ablashi, G., de Thé, G., Lenoir, G. & Pearson, G.R. (eds) pp. 777–790. John Libbey Furotext Ltd.

JEANNEL, D., HUBERT, A., DE VATHAIRE, F., ELLOUZ, R., CAMOUN, M., BEN SALEN, M., SANCHO-GARNIER, H. & DE THE, G. (1990). Diet, living conditions and nasopharyngeal carcinoma in Tunisia: a case–control study. *Int. J. Cancer*, **46**, 421–425.

LANIER, A.P., BENDER, T.R., BLOT, W.J., FRAUMENI, J.F. & HURLBERT, W.B. (1976). Cancer incidence in Alaska natives. *Int. J. Cancer*, **18**, 409–412.

KAI-TAI, Y., PENG-NIAN, W. & JI-WEN, J. (1987). The role of promotion in the carcinogenesis of nasopharyngeal carcinoma. In *Cancer of the Liver, Esophagus, and Nasopharynx*, Wagner, G. & Zhang, Y.H. (eds) pp. 187–193. Springer: Berlin.

LEVINE, P.H., HUANG, A.T., WEILAND, L., HILDESHEIM, A., BRIZEL, D., BOYCE COLE, T., FISHER, S.R., PANELLA, T.J. & JIAN, J. (1990). Nasopharyngeal carcinoma: 1990. In *Epstein–Barr Virus and Human Disease*, Ablashi, D., Huang, A., Pagano, J., Pearson, G. & Yang, C. (eds) pp. 313–330. Humana Press: Clifton, NJ.

LIN, T.M., CHEN, K.P., LIN, C.C., HSU, M.M., CHIANG, T.C., JUNG, P.F. & HIRAYAMA, T. (1973). Retrospective study on nasopharyngeal carcinoma. *J. Natl. Cancer Inst.*, **51**, 1403–1408.

LU, S.J., DAY, N.E., DEGOS, L., LEPAGE, V., WANG, P.C., CHAN, S.H., SIMONS, M., McKNIGHT, B., EASTON, D., ZENG, Y. & DE THE, G. (1990). Linkage of a nasopharyngeal carcinoma susceptibility locus to the HLA region. *Nature*, **346**, 470–471.

MUIR, C., WATERHOUSE, J., MACK, T., POWELL, J. & WHELLEN, S. (eds) (1987). *Cancer Incidence in Five Continents*, Vol. 88, IARC: Lyon.

NING, J.P., YU, M.C., WANG, Q.S., HENDERSON, B.E. (1990). Consumption of salted fish and other risk factors for nasopharygeal carcinoma (NPC) in Tianjin, a low-risk region for NPC in the People's Republic of China. *J. Natl Cancer Inst.*, **82**, 291–296.

PARKIN, D.M. (ed.) (1986). *Cancer Occurrence in Developing Countries*. Vol. 75, IARC: Lyon.

POIRIER, S., OSHIMA, H., DE THE, G., HUBERT, A., BOURGADE, M.C. & BARTSCH, H. (1987). Volatile nitrosamine levels in common foods from Tunisia, South China and Greenland, high risk areas for nasopharyngeal carcinoma. *Int. J. Cancer*, **39**, 293–296.

SHANMUGARATNAM, K., TYE, C.Y., GOH, E.H. & CHIA, K.B. (1978). Etiological factors in nasopharygeal carcinoma: a hospital based, retrospective, case-control, questionnaire study. In *Nasopharyngeal Carcinoma: Etiology and Control*, Vol. 20, de Thé, G. & Ito, Y. (eds) pp. 199-212. IARC: Lyon.

SHAO, Y.M., POIRIER, S., OSHIMA, H., MALAVIELLE, C., ZENG, Y., DE THE, G. & BARTSCH, H. (1988). Epstein-Barr virus activation in Raji cells by extracts of preserved foods from high risk areas for nasopharyngeal carcinoma. *Carcinogenesis*, **9**, 1455-1457.

WATERHOUSE, J.A.H., MURI, C., SHANMUGARATNAM, K. & 3 others (1982). *Cancer Incidence in Five Continents*, Vol. 42. IARC: Lyon.

YU, M.C., HO, J.H.C., LAI, S.H. & HENDERSON, B.E. (1986). Cantonese-style salted fish as a cause of nasopharyngeal carcinoma: report of a case-control study in Hong Kong. *Cancer Res.*, **46**, 956-961.

YU, M.C., MO, C.C., CHONG, W.X., YEH, F.S. & HENDERSON, B.E. (1988). Preserved foods and nasopharyngral carcinoma: a case-control study in Guangxi, China. *Cancer Res.*, **48**, 1954-1959.

YU, M.C., HUANG, T.B., HENDERSON, B.E. (1989). Diet and nasopharyngeal carcinoma: a case-control study in Guangzhou, China. *Int. J. Cancer*, **43**, 1077-1082.

YU, M.C., GARABRANT, D.H., HUANG, T.B., HENDERSON, B.E. (1990). Occupational and other non-dietary risk factors for nasopharyngeal carcinoma in Guangzhou, China. *Int. J. Cancer*, **45**, 1033-1039.

ZENG, Y., ZHONG, J.M. & MIAO, X.C. (1983). Epstein-Barr virus early antigen induction in Raji cells by Chinese medicinal herbs. *Intervirology*, **19**, 201-204.